# Transmission of X-linked Ovarian Cancer: Characterization and Implications

**DOI:** 10.3390/diagnostics10020090

**Published:** 2020-02-07

**Authors:** John Lewis Etter, Kirsten Moysich, Shaun Kohli, Shashikant Lele, Kunle Odunsi, Kevin H. Eng

**Affiliations:** 1 Department of Cancer Prevention and Control, Roswell Park Comprehensive Cancer Center, Buffalo, NY 14263, USA; 2 Department of Epidemiology and Environmental Health, State University of New York at Buffalo, Buffalo, NY 14263, USA; 3 Jacobs School of Medicine and Biomedical Sciences, State University of New York at Buffalo, Buffalo, NY 14263, USA; 4 Department of Cancer Genetics and Genomics, Roswell Park Comprehensive Cancer Center, Buffalo, NY 14263, USA; 5 Department of Biostatistics and Bioinformatics, Roswell Park Comprehensive Cancer Center, Buffalo, NY 14263, USA; 6 Department of Gynecologic Oncology, Roswell Park Comprehensive Cancer Center, Buffalo, NY 14263, USA

**Keywords:** ovarian cancer, hereditary cancer, familial cancer, X-linked disease

## Abstract

We recently reported evidence that a strong, BRCA-independent locus on the X-chromosome may contribute to ovarian cancer predisposition in families ascertained from the Familial Ovarian Cancer Registry (Buffalo, NY, USA). While it has been estimated that approximately 20% of all ovarian cancer cases are hereditary, it is possible that a significant proportion of cases previously believed to be sporadic may, in fact, be X-linked. Such X-linked disease has a distinct pattern; it implies that a father will necessarily pass a risk allele to each of his daughters, increasing the prevalence of cancers clustered within a family. X-chromosome inactivation further influences the expression of X-linked alleles and may represent a novel target for screening and therapy. Herein, we review the current literature regarding X-linked ovarian cancer and interpret allele transmission-based models to characterize X-linked ovarian cancer and develop a framework for clinical and epidemiological familial ascertainment to inform the design of future studies.

## 1. Introduction

Ovarian cancer is the leading cause of gynecologic cancer-related death among females in the United States with nearly 14,000 estimated deaths in 2019. Among the 22,530 estimated new cases of ovarian cancer in 2019, only 47.6% are expected to survive at least five years. Survival, however, is highly dependent on stage at diagnosis. While those diagnosed with localized disease experience a favorable five-year survival rate of 92.4%, those diagnosed with distant disease experience a five-year survival rate of just 29.2%. Despite this, 59% of ovarian cancer patients are still diagnosed with distant disease according to the most recent National Cancer Institute Surveillance Epidemiology and End Results Program statistics [1].

Identification of individuals at high-risk for developing ovarian cancer is thus critical to improving early detection. Family history of ovarian cancer has been well-established to increase a female’s risk of ovarian cancer with a first- or second-degree affected relative increasing an individual’s risk 3.6- or 2.8-fold, respectively, and two affected family members increasing an individual’s risk 5-fold [2]. It has been previously estimated that hereditary ovarian cancer accounts for at least 20% of all ovarian cancer cases in the United States, with approximately 65–85% and 10–15% of these cases attributable to BRCA mutations or Lynch syndrome, respectively [3]. Knowledge of these hereditary cases empowers physicians to identify high-risk families and engage them in enhanced screening and risk-reduction dialogue to ultimately reduce ovarian cancer incidence and mortality.

Our research group recently reported evidence from the Familial Ovarian Cancer Registry (FOCR, Buffalo, NY) of a novel X-linked ovarian cancer susceptibility gene variant that may be causing previously unrecognized cases of hereditary ovarian cancer independent of BRCA [4]. We posit that, under current workflows, a substantial and specific proportion of X-linked ovarian cancer cases are falsely characterized as non-hereditary and that high-risk family members of these X-linked cases consequently fail to receive life-saving early screening and counseling.

In this follow-up report, we review what is currently known regarding the X-chromosome and ovarian cancer and present a transmission model-based framework to inform how clinicians and researchers might identify, study and manage families at high risk for X-linked ovarian cancer.

### The X-Chromosome and Cancer

The X-chromosome is one of two human sex chromosomes responsible for determining the sex of an individual. While males possess both one X- and one Y-chromosome, females lack a Y-chromosome and instead carry two X-chromosomes. As a result, the X-chromosome follows a pattern of transmission unique from that of autosomes. Unlike autosomal alleles, which are passed from parent to offspring with 1/2 probability, irrespective of the sex of the parent or the offspring, transmission of X-linked alleles varies by the sex of both the parent and the offspring. Mothers pass an X-linked allele to a son or a daughter with 1/2 probability, while fathers pass an X-linked allele to all daughters with 100% probability and never to sons.

To presumably achieve equal X-chromosome gene dosage with males, females undergo X-chromosome inactivation (XCI), an epigenetic mechanism by which one X-chromosome is randomly and permanently inactivated in each cell during early embryogenesis via the expression of the X-inactive—specific transcript (Xist), a long non-coding RNA that coats and prevents transcription of the X-chromosome in cis. Each subsequent daughter cell retains the XCI pattern of its parent cell. Inactivated X-chromosomes can be visualized histologically as small, aggregated, dark-staining, heterochromatic structures known as Barr bodies within the nuclei of female somatic cells. Nevertheless, approximately 15–25% of genes on the X-chromosome have been shown to escape X-chromosome inactivation either by their location within the pseudoautosomal regions (PAR), distal regions of the p and q arms of the X-chromosome that are homologous with the Y-chromosome and inherited autosomally, or through other escape mechanisms outside of the PAR [5]. Genes outside the PAR that escape XCI are biallelically expressed in females, whereas those within the PAR are biallelically expressed in both females and males. It has been suggested that genes outside the PAR that escape XCI may be responsible for an observed sex bias in certain cancers [6].

The X-chromosome is known to contain many genes related to cancer [7]. One such group of genes are the “cancer-testis” genes, which, although only normally expressed in adult tissue by the testes, have been observed to also be expressed by many tumors, including those of the ovary [8,9]. Moreover, loss of heterozygosity (LOH) and skewing of XCI at specific loci on the X-chromosome have implicated these regions as potential sites for novel recessive X-linked tumor suppressors [7]. Cancer cells traditionally silence tumor suppressors via a two-hit process whereby LOH, or the “first-hit”, is followed by a sporadic “second-hit” resulting in no functional copies of the tumor suppressor [10]. Skewed XCI occurs when the ratio of inactivated paternal and maternal X-chromosomes deviates from an equilibrium of 1:1 due to genetic factors, selection or chance. Such skewed XCI may lead to functional LOH in all affected cells rendering them susceptible to a “second-hit” [11]. X-linked LOH has been observed in upwards of 40% of ovarian cancers [12,13] and has been associated with increased risk of progression, higher tumor grade and cisplatin resistance [14]. Skewed XCI has also been reported to be positively associated with BRCA mutations [11,15]; however, several subsequent studies have failed to corroborate these results [16,17,18].

It has been reported, albeit inconsistently [15], that a greater proportion of ovarian cancer cases exhibit skewed XCI than controls [11]. Likewise, ovarian cancer cell lines have been observed to lose the inactivated X-chromosome (Xi), duplicate the active X-chromosome (Xa) and re-activate Xi [14,19], and ovarian tumors have been observed to exhibit apparent dysregulation of X-chromosome inactivation, as evidenced by a loss of Barr bodies in cancer cells [19,20]. A recent study found that the majority (52%) of ovarian tumors exhibited alterations to the X-chromosome, and 96% of the remaining, structurally unaltered tumors exhibited skewed XCI [21]. A follow-up study identified a molecular subgroup of ovarian tumors with dysregulated XCI that was associated with shorter time to recurrence and overall survival time [22].

It has long been observed that sisters of ovarian cancer patients are at greater risk for ovarian cancer than their mothers [23], a phenomenon consistent with X-linkage. Using pedigree data from the FOCR, our research group was able to recapitulate these findings with ovarian cancer rates among mothers and sisters of ovarian cancer cases of 35% and 66%, respectively. After restricting to ovarian cancer cases with exactly one affected grandmother, we observed that these individuals were twice as likely to have a paternal grandmother (28.4%, 95% CI: 22.8–34.8%) affected with ovarian cancer than a maternal grandmother (13.9%, 95% CI: 11.4–16.8%). These rates were consistent with an X-linked transmission model. Those with affected paternal grandmothers were significantly more likely to develop earlier onset ovarian cancer than those with affected maternal grandmothers (HR 1.59, 95% CI: 1.12–2.25) [4]. Using exome-sequencing data from 159 self-reported BRCA-negative ovarian cancer cases in the FOCR, an age-of-onset analysis identified a missense variant rs176026 of MAGEC3 at Xq27.2 that met X-chromosome-wide significance. 15.2% (21/159) of sequenced cases were carriers of this risk allele with a minor allele frequency of 6.9% (22/318). Carriers heterozygous for the risk allele experienced an overall 6.7-year earlier onset of ovarian cancer. When stratified by affected family members, heterozygous carriers with only an affected sister experienced a 11.0-year earlier onset of ovarian cancer.

## 2. Materials and Methods

In order to better understand the familial inheritance of an X-linked ovarian cancer risk allele with respect to an autosomal allele, we considered a selectively neutral two-locus model with discrete generations conditional on a pre-specified ancestral genotype. These two loci were assumed to lie on an autosomal and a sex chromosome. Let {A, a} be alleles for the autosomal locus where A is a disease risk allele and a is a non-risk allele. Let {X, x, y} be alleles for the sex chromosome locus where X indicates the disease risk allele and y indicates inheritance of a father’s Y-chromosome by his son. Forgoing homozygous carriers, there are eight genotypes of interest spanning both sexes: {AaXx, Aaxx, aaXx, aaxx} for females and {AaXy, Aaxy, aaXy, aaxy} for males.

The initial founding case in these families was a female who carried both risk alleles (AaXx) and randomly generated offspring according to a Poisson law with expectation λ = 2.4 (the average children per household in the US population). The genotypes for these offspring were generated assuming alleles assort independently and that mates into this family are non-carriers at both loci (aaxx or aaxy). Following these simple assortment rules, Table 1 is the transition matrix P=(p_ij), where entry p_ij is the probability that a person with genotype i has an offspring with genotype j. As in the usual Markov chain model, the rows of this transition matrix sum to 1. This model tracks and tabulates the number of alleles in each generation and therefore does not make any assumptions for penetrance, XCI or impact on fitness.

Next, we considered family structure and size in our analysis of X-linked and autosomal risk allele transmission via three parallel simulation models using the same transmission probabilities from Table 1. Each model differed by the genotype of its founding female: aaXx (X-linked), Aaxx (autosomal) and AaXx (two-locus). Again, each mating pair randomly generated offspring according to a Poisson law with expectation λ = 2.4. After simulating 10,000 families for each model, we tabulated relationship pairs of third-generational female risk allele carriers and their carrier family members.

## 3. Results

**Figure 1:**
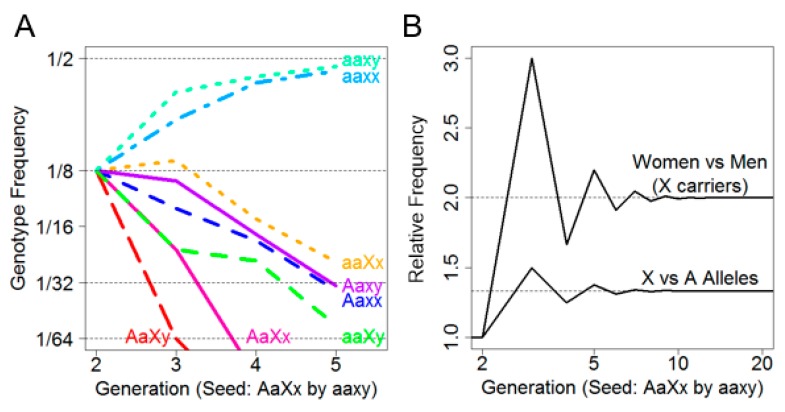
(**A**) (left): Transition probabilities over five generations showing decay towards the persistent non-risk genotypes; and (**B**) (right): The sex ratio of X carriers showing a limiting behavior due to carrier fathers.

Because no new risk alleles entered these families, the risk alleles were transient; at five generations (P^5), the probability of carrying either risk allele was 3.1%. Figure 1A depicts the frequency of each of the eight genotypes over five generations. It was observed that the non-risk genotypes aaxx and aaxy predominated by the second generation and increased in frequency with each subsequent generation until convergence at 1/2 frequency by approximately the fifth generation. Intriguingly, among the risk allele-carrying genotypes, aaXx was persistently the most frequent. Not only were female X-linked risk allele carriers more frequent than male X-linked risk allele carriers, they were also more frequent than female autosomal risk allele carriers.

This is further illustrated in Figure 1B, where the ratio of female (aaXx) to male (aaXy) X-linked risk allele carriers by generation was plotted (above) as well as the ratio of female X-linked risk allele carriers (aaXx) to female autosomal risk allele carriers (Aaxx) by generation (below). We noted that while the overall sex ratio remained 1:1 in every generation, the ratio of females who carried only the X allele (aaXx) versus males (aaXy) converged on two after approximately five generations, indicating that females are more likely to carry an X-linked risk allele than males.

Moreover, as the number of generations increased, the rate of female Xx carriers to female Aa carriers converged on 4/3. This suggested that, under assumptions of no selective force and equivalent founder generations, an X-linked risk allele is expected to be 33% more prevalent than an autosomal risk allele. Finally, there appeared to be an oscillation in female and male X-linked risk allele carriers due to a lack of father–son transmission.

Tabulated relationship pairs of third generational female carriers are found in Table 2 for each of the three models (X-linked, autosomal and two-locus). As expected, we observed that X-linked father–daughter pairs occurred twice as frequently as X-linked mother–daughter pairs, autosomal mother–daughter pairs and autosomal father–daughter pairs. Likewise, we observed that X-linked father–daughter pairs were significantly more likely to have a carrier sister than X-linked mother–daughter pairs, autosomal mother–daughter pairs and autosomal father–daughter pairs given that a father must pass his X-chromosome to all of his daughters.

While sister–sister carrier pairs were more frequent in the X-linked model than the autosomal model, so too were father–only daughter pairs. Paternal aunt–niece pairs were also more common in the X-linked model than the autosomal model. In the two-locus model (founder genotype AaXx), there were a greater number of pairs for all relationship types due to the presence of twice as many founding risk alleles.

We also observed that, at the third generation, an X-linked risk allele was significantly more likely to be paternally inherited than maternally inherited as the number of carrier daughters increased (Table 3). This trend was unique to X-linkage and was thus only present when an X-linked risk allele was transmitted, as seen in the X-linked and two-locus models.

## 4. Discussion

With mounting evidence implicating the X-chromosome in ovarian carcinogenesis, future studies designed to better understand the clinical, epidemiological and molecular characteristics of X-linked ovarian cancer will rely on investigators’ ability to identify cases likely (and unlikely) to be X-linked. Such identification can be informed by the following basic corollaries derived from an understanding of X-linked transmission and XCI, as well as our analysis results.

**Figure 2:**
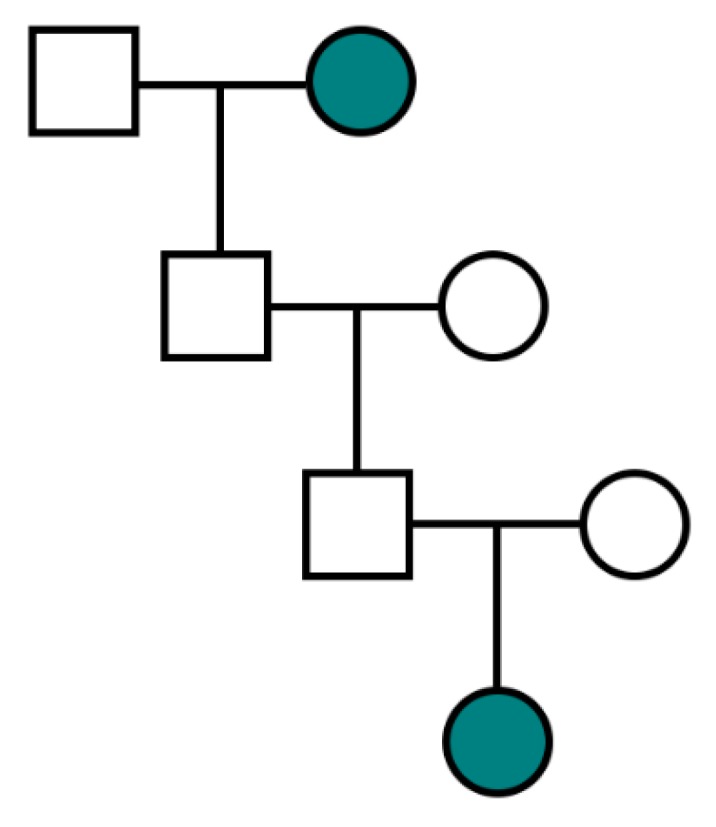
Pedigree structure incompatible with X-linked ovarian cancer transmission due to two generations of intervening males. Teal represents an ovarian cancer case.

Firstly, X-linked ovarian cancers are more likely to be transmitted via a paternal lineage, as demonstrated in Table 2. When considering a proband with X-linked ovarian cancer, it is expected that paternal grandmothers will be twice as likely to have had ovarian cancer than maternal grandmothers. This is consistent with observations in the FOCR, where 28.4% of paternal grandmothers were affected as compared with 13.9% of maternal grandmothers. Secondly, all daughters of paternal X-linked risk allele carriers will also be carriers such that the greater number of affected sisters in a family, the greater the likelihood of X-linkage, as illustrated by Table 3. Lastly, a pedigree with multiple familial ovarian cancers separated by two sequential intervening males is not compatible with X-linkage given that a father must pass a Y-chromosome to his son rather than an X-chromosome (Figure 2).

**Figure 3:**
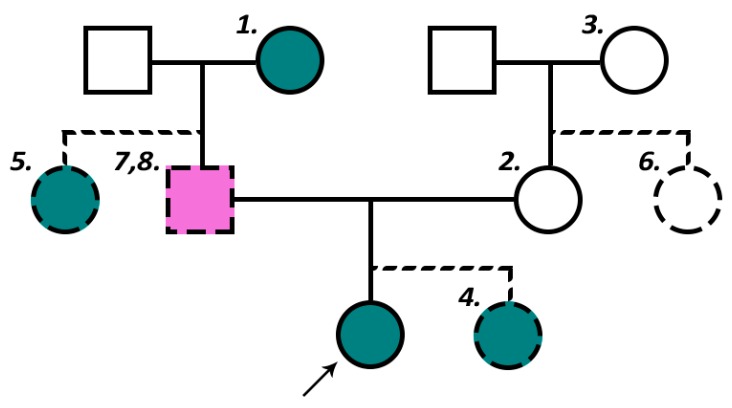
Pedigree structure enriched for X-linked ovarian cancer. Teal represents an ovarian cancer case. Purple represents any cancer case. Numbers correspond to numerated criteria from text. Note that, in this example, dashed lines represent elective criteria.

Considering these corollaries, we can appreciate a three-generation pedigree structure enriched for X-linked ovarian cancer (Figure 3) defined by the following criteria:

(1) The proband’s paternal grandmother is affected;
(2) The proband’s mother is unaffected; and
(3) The proband’s maternal grandmother is unaffected.

When applicable, additional elective criteria include the following:

(4) At least one of the proband’s sisters are affected;
(5) At least one of the proband’s paternal aunts are affected; and
(6) The proband’s maternal aunts are unaffected.

If more than three generations of pedigree information are available, an additional criterion is the following:

(7) There are no occurrences of two sequential generations of intervening males in a transmission lineage (Figure 2).

Considering the impact of X-linked penetrance and sex-specific disease reporting bias, investigators may also wish to consider the elective criterion:

(8) A history of cancer in the proband’s father.

Consideration of XCI may also be informative in the identification of X-linked ovarian cancer cases. Females typically undergo random XCI such that, on average, half of their maternally and paternally inherited X-chromosomes are active. This means that an X-chromosome harboring a given susceptibility allele, denoted X* for this discussion, would be expected to be active, on average, in only 50% of a heterozygous female’s cells under completely random XCI. Therefore, at an individual level, females carrying X* are, on average, at 50% lesser risk of developing a corresponding X*-phenotype than males carrying X*, assuming completely random XCI. On a population level, however, Figure 1B demonstrated that females are twice as likely to carry an X-linked risk allele than males after five generations. Females are thus twice as likely to carry X* but half as likely to express the corresponding X*-phenotype due to XCI. Disregarding additional epigenetic mechanisms, this would imply that males and females are equally likely, on a population level, to express a X*-phenotype after approximately five generations. It has been reported, however, that 15–25% of genes on the X-chromosome, including MAGEC3 [6], escape XCI [5]. Such escape allows for biallelic expression and subsequent rescuing of X-linked genes, thereby further reducing X*-carrying females’ risk of expressing an X*-linked phenotype relative to X*-carrying males.

It follows that males may be expected to experience greater X-linked penetrance than females. Consistent with this expectation and the assumptive existence of a cancer-causing X-linked locus escaping XCI, age- and sex-adjusted cancer incidence rates are statistically significantly greater in males than females for the majority of non-sex-specific cancer types [24]. While the majority of studies investigating X-linked ovarian cancer have focused primarily on females with ovarian cancer, there is no evidence that such X-linkage would be unique to females or ovarian cancer. Conversely, there exists compelling evidence that an X-linked cancer-causing gene would affect males more than females, assuming, of course, that its expression is not exclusive to tissue types from which ovarian cancers arise. Considering this, along with the corollary that X-linked cancers are more likely to be transmitted via a paternal lineage, it may be sensible for investigators to consider familial male cancers in their study of X-linked ovarian cancer. Our research group has previously reported evidence for associations consistent with X-linkage between familial ovarian cancer and both prostate and testicular cancers [4,25]. Investigators may thus consider modifying the pedigree structure enriched for X-linkage in Figure 3 to include an additional elective criterion of history of cancer in the proband’s father (criterion 8).

XCI may either promote or suppress the cellular expression of an X-linked susceptibility allele depending on whether X* is activated or inactivated. On a larger scale, skewed XCI can promote or suppress a given X-linked phenotype depending on the global proportion of X* inactivated. Those with skewed XCI toward greater X* activation are thus at an increased predisposition to develop a given X-linked disease than those with non-skewed XCI or those with skewed XCI toward greater X* inactivation. In contrast, those with greater X* inactivation would be at lesser predisposition for developing a given X-linked disease than those with non-skewed XCI or those with greater X* activation.

Although it has been shown that skewed XCI at a 65% threshold can occur in the general female population simply due to chance, it is far less commonly observed in the general female population at higher skewing thresholds such as that of 85% [26]. Moreover, it has been reported that a greater proportion of ovarian cancer cases exhibit skewed XCI at a threshold of 75% than controls [11]. As it can be reasoned that ovarian cancer cases with skewed XCI may be more likely to carry an X-linked ovarian cancer susceptibility allele than cases with non-skewed XCI, skewed XCI should be systematically evaluated as a marker for X-linked ovarian cancer.

## 5. Conclusions

An enhanced ability to identify X-linked ovarian cancer will serve to empower studies aimed at better understanding the genetic and epigenetic underpinnings of these cancers. This knowledge bears potentially significant implications for screening, risk reduction and treatment. Knowledge of specific X-linked risk alleles could enable genetic detection of carriers at increased risk for developing X-linked ovarian cancer. These carriers could be engaged in earlier education, counseling and screening programs to promote early detection as well as discussions to consider potential prophylactic risk-reducing procedures. Ovarian cancer patients and their families could also benefit from knowledge of specific X-linked risk alleles. As it is likely that a proportion of ovarian cancers previously believed to be sporadic are, in fact, X-linked, family members of X-linked cases could be referred into medical genetics programs for screening and counseling. Cases themselves could also benefit from potential immunotherapies and genetic therapies targeting these X-linked risk alleles. A greater understanding of the molecular mechanisms responsible for XCI could also inform the development of novel XCI-driven therapies. Considering that many X-linked genes escape XCI, it may be possible to exploit these mechanisms to preferentially reactivate the wild-type X-linked alleles and rescue cells from skewing-induced LOH. It is our hope that the information and framework described herein may inform the design of future X-linked ovarian cancer studies to further advance the field toward these potential preventative and therapeutic innovations to ultimately improve ovarian cancer incidence and mortality.

## Figures and Tables

**Table 1:** Two-locus transition probabilities (multiples of 1/8) between parent (row) and offspring (column).

	Next Generation								
		AaXx	Aaxx	aaXx	aaxx	AaXy	Aaxy	aaXy	aaxy
**Current Generation**	AaXx	1	1	1	1	1	1	1	1
	Aaxx	0	2	0	2	0	2	0	2
	aaXx	0	0	2	2	0	0	2	2
	aaxx	0	0	0	4	0	0	0	4
	AaXy	2	0	2	0	0	2	0	2
	Aaxy	0	2	0	2	0	2	0	2
	aaXy	0	0	4	0	0	0	0	4
	aaxy	0	0	0	4	0	0	0	4

**Table 2:** Familial relationships among third-generation females who carry risk alleles (10,000 simulated families, λ =2.4).

Relationship	X-linked	Autosomal	Two-Locus
Mother–daughter	3608	3609	6136
Only daughter	1094	1077	1882
Has carrier sister	1642	1636	3104
Non-carrier sister	872	896	1150
Father–daughter	7132	3661	9120
Only daughter	2164	1084	2742
Has carrier sister	4968	1639	5906
Non-carrier sister	0	938	472
Sister–sister	6610	3275	8010
Maternal Aunt–niece	2124	2107	5086
Paternal Aunt–niece	4188	2054	7923

**Table 3:** Simulated frequencies of maternal and paternal lineages by number of carrier daughters.

	X-Linked		Autosomal		Two-Locus	
Number of Carrier Daughters	Maternal	Paternal	Maternal	Paternal	Maternal	Paternal
1	1966	2164	1973	2022	3032	3214
2	603	1285	573	588	1024	1596
3	118	526	144	131	306	566
4	14	135	13	15	28	194
5	4	38	0	2	4	38
6	1	15	1	0	1	6
7	0	0	0	0	0	2
